# Pulmonary miliary sarcoidosis masquerading the reactivation of tuberculosis 30 years later

**DOI:** 10.1002/ccr3.7624

**Published:** 2023-06-23

**Authors:** Takashi Okuma, Reimi Mizushima, Yukihisa Takeda, Kazutoshi Toriyama, Yusuke Watanabe, Hiroyuki Nakamura, Kazutetsu Aoshiba

**Affiliations:** ^1^ Department of Respiratory Medicine Tokyo Medical University Ibaraki Medical Center Ibaraki Japan; ^2^ Department of Respiratory Medicine Tokyo Medical University Hospital Tokyo Japan; ^3^ Department of Infection Prevention and Control Tokyo Medical University Hospital Tokyo Japan

**Keywords:** granuloma, high‐resolution computed tomography, miliary tuberculosis, sarcoidosis

## Abstract

**Key Clinical Message:**

Sarcoidosis may occur after treatment with pulmonary tuberculosis and requires differential diagnosis from tuberculosis reactivation. Miliary sarcoidosis should be promptly differentiated from miliary tuberculosis associated with high mortality.

**Abstract:**

Clinical, histological, and radiological similarities between sarcoidosis and tuberculosis render differential diagnosis challenging. The association between these two diseases has long been discussed, although the coexistence or subsequent occurrence of tuberculosis and sarcoidosis is rare. We report a case of miliary sarcoidosis that developed 30 years after tuberculous pleurisy treatment. Sarcoidosis may occur after treatment with pulmonary tuberculosis and requires differential diagnosis from tuberculosis reactivation. Although miliary sarcoidosis is uncommon, it should be promptly differentiated from miliary tuberculosis associated with high mortality. This study reignites the debate on the causal association between tuberculosis and sarcoidosis.

A 61‐year‐old asymptomatic woman presented for a routine medical checkup. Thirty years ago, she received antituberculous drug treatment for pleural fluid culture‐positive left tuberculous pleurisy. A chest X‐ray taken 1 year ago showed unremarkable findings except for an old left pleural scar. However, this year, her chest X‐ray showed tiny nodules distributed throughout the bilateral lungs with upper lobe predominance (Figure 1A). High‐resolution computed tomography image of the chest revealed diffuse micronodules dispersed in a random miliary pattern (Figure 1B). These micronodules were not prominent on the bronchovascular bundle and interlobular septa. Hilar and mediastinal lymph nodes were unremarkable. Initially, reactivation of tuberculosis was suspected with miliary lung opacities resulting from hematogenous dissemination of the tubercle bacilli. An alternative diagnosis included a metastatic lung tumor. Therefore, bronchoscopy with bronchoalveolar lavage and lung biopsy was performed. Mycobacterial examination of the sample using acid‐fast bacilli smear and culture and polymerase chain reaction tests for *Mycobacterium tuberculosis* and *Mycobacterium avium* complex were negative. Histopathological examination of the biopsied lung tissue disclosed the presence of noncaseating granuloma (Figure 2). No malignancy was identified. Laboratory blood test results showed normal parameters except for elevated angiotensin‐converting enzyme (27.8 U/L; normal range: 8.3–21.4 U/L), lysozyme (19.6 μg/mL; normal range: 5–10 μg/mL), and soluble interleukin‐2 receptor (1470 U/mL; normal range: 157–474 U/mL). Based on the above findings, the patient was diagnosed with miliary sarcoidosis that developed 30 years after treatment with tuberculosis. As the patient's condition was stable, corticosteroid treatment was not initiated. During the 3‐montht follow‐up, she remained asymptomatic without progression of radiologic abnormalities. Although very rare (<1% of sarcoidosis cases), miliary pattern of sarcoidosis is observed as diffuse micronodules located in a random distribution resembling miliary tuberculosis and metastatic lung tumor, without the classic peribronchovascular and perilymphatic location typical of sarcoidosis. In miliary sarcoidosis, the lung opacities tend to have an upper lobe predominance, as in this case. In this context, Rajagopala et al. coined the term “pseudomiliary” because a majority of “miliary” sarcoidosis cases had a perilymphatic pattern.^1^ However, they indicated that 34.6% of reported cases could be classified as a “true miliary” pattern with only very subtle perilymphatic nodules, as in our case. They also reported that approximately half of “miliary” or “pseudomiliary” sarcoidosis cases had a preceding or concurrent tuberculosis.^1^ Tuberculosis and sarcoidosis are chronic granulomatous diseases involving the lungs and other organs. A causal association between mycobacterial infection and sarcoidosis has long been discussed. For example, case report studies, including the current report, described that tuberculosis and sarcoidosis occurred concomitantly or subsequently. Remnant *Mycobacterium tuberculosis* catalase peroxidase has been identified as a possible antigen of the immune response in sarcoidosis.^2^ A meta‐analysis study also revealed that 26% of biopsies from patients with sarcoidosis was positive for mycobacterial genes.^3^ These studies suggest that mycobacterial antigens may elicit an immune response leading to sarcoidosis in a susceptible host. In our case, sarcoidosis developed three decades after treatment of tuberculous pleurisy, suggesting chronic presence of tuberculous antigens after antituberculosis therapy.

**FIGURE 1:**
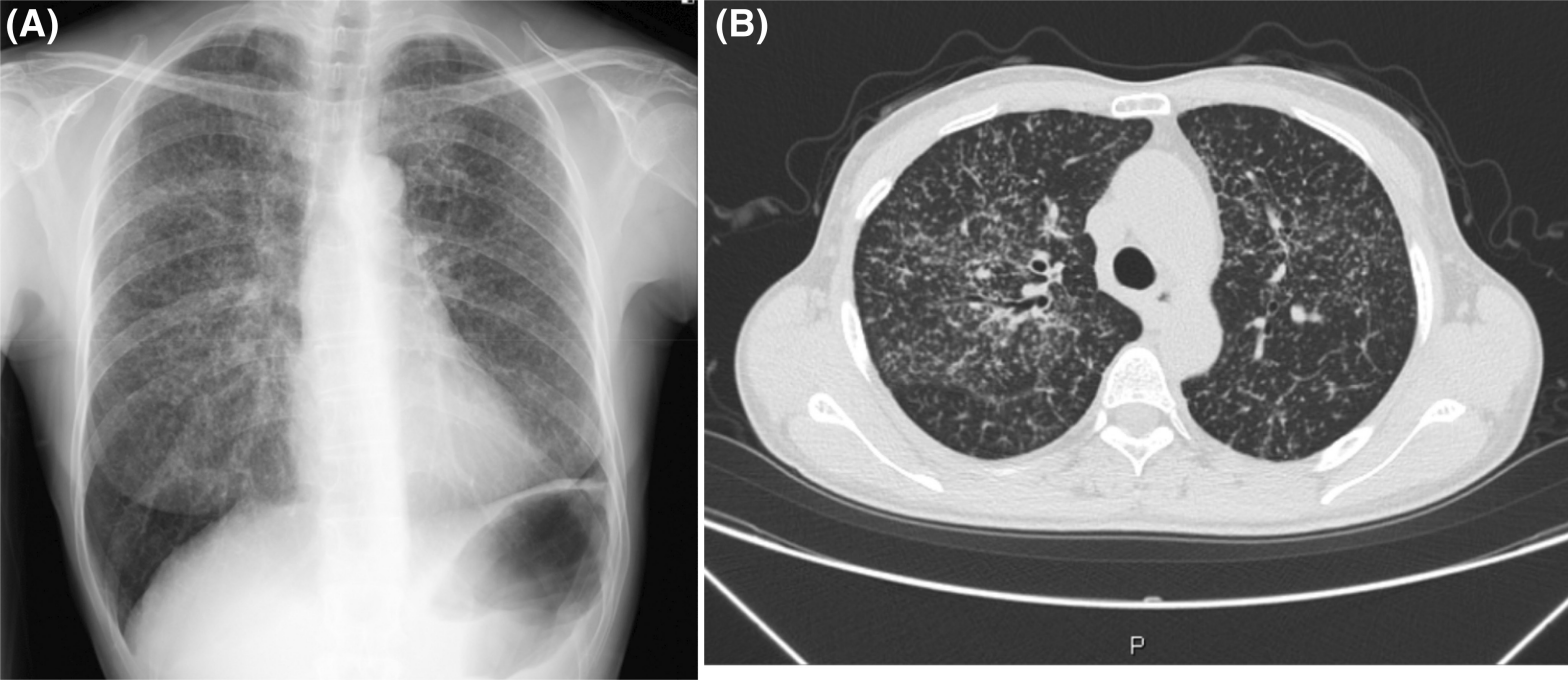
Radiographic images of the chest. (A) Chest X‐ray demonstrating tiny nodules scattered throughout the bilateral lungs with upper lobe predominance. (B) A high‐resolution computed tomography image displaying diffuse miliary opacities in a random miliary pattern as well as very subtle perilymphatic micronodules.

**FIGURE 2:**
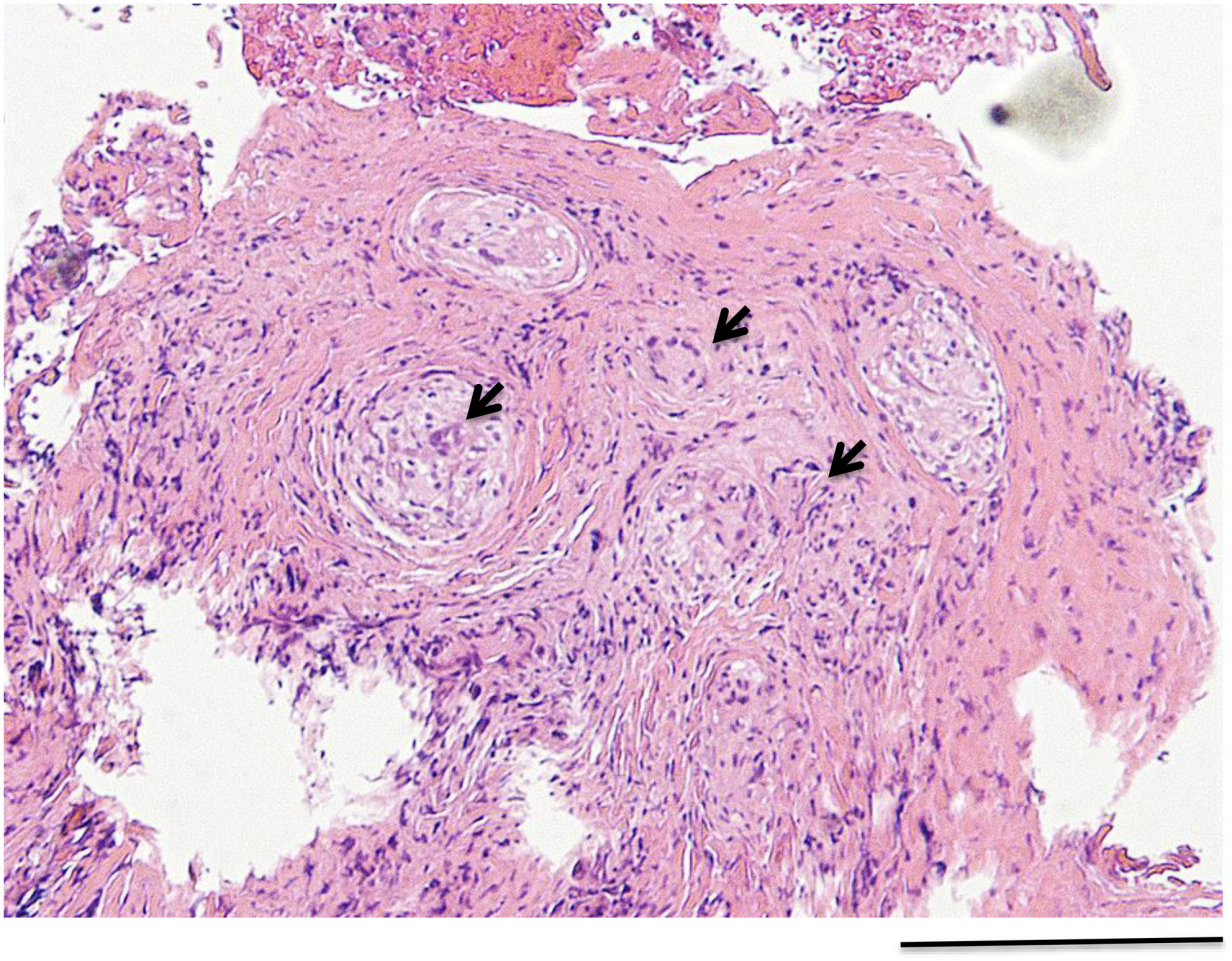
Hematoxylin and eosin‐stained lung biopsy specimens showing noncaseating epithelioid granuloma with occasional Langhans giant cells (arrows). Scale bar: 50 μm.

## AUTHOR CONTRIBUTIONS

**Takashi Okuma:** Writing – original draft. **Reimi Mizushima:** Writing – review and editing. **Yukihisa Takeda:** Writing – review and editing. **Kazutoshi Toriyama:** Writing – review and editing. **Yusuke Watanabe:** Writing – review and editing. **Hiroyuki Nakamura:** Writing – review and editing. **Kazutetsu Aoshiba:** Conceptualization; writing – original draft.

## FUNDING INFORMATION

No sources of funding were used.

## CONFLICT OF INTEREST STATEMENT

The authors have no conflicts of interest to declare.

## CONSENT STATEMENT

Written consent from the patient was obtained for submission and publication of the case details and images from the patient. This case report meets the standards of Tokyo Medical University ethical committee.

## Data Availability

The data supporting the findings of this study are available from the corresponding author upon request; they are not publicly available due to privacy or ethical restrictions.
